# Glutathione S-transferase L1 multiplex serology as a measure of cumulative infection with human papillomavirus

**DOI:** 10.1186/1471-2334-14-120

**Published:** 2014-03-03

**Authors:** Hilary A Robbins, Yan Li, Carolina Porras, Michael Pawlita, Arpita Ghosh, Ana Cecilia Rodriguez, Mark Schiffman, Sholom Wacholder, Troy J Kemp, Paula Gonzalez, John Schiller, Douglas Lowy, Mark Esser, Katie Matys, Wim Quint, Leen-Jan van Doorn, Rolando Herrero, Ligia A Pinto, Allan Hildesheim, Tim Waterboer, Mahboobeh Safaeian

**Affiliations:** 1 Division of Cancer Epidemiology and Genetics, National Cancer Institute, NIH, Rockville, Maryland, USA; 2 Joint Program for Survey Methodology, University of Maryland, College Park, Maryland, USA; 3 Proyecto Epidemiológico Guanacaste, Fundación INCIENSA, Guanacaste, Costa Rica; 4 German Cancer Research Center (DKFZ), Heidelberg, Germany; 5 Public Health Foundation of India, New Delhi, India; 6 HPV Immunology Laboratory, SAIC-Frederick Inc., Frederick National Laboratory for Cancer Research, Frederick, Maryland, USA; 7 International Agency for Research on Cancer, Lyon, France; 8 Center for Cancer Research, National Cancer Institute, NIH, Bethesda, Maryland, USA; 9 MedImmune, Gaithersburg, Maryland, USA; 10 PPD Vaccines and Biologics Center of Excellence, Wayne, Pennsylvania, USA; 11 DDL Diagnostic Laboratory, Rijswijk, Netherlands

## Abstract

**Background:**

Several assays are used to measure type-specific serological responses to human papillomavirus (HPV), including the bead-based glutathione S-transferase (GST)-L1 multiplex serology assay and virus-like particle (VLP)-based ELISA. We evaluated the high-throughput GST-L1, which is increasingly used in epidemiologic research, as a measure of cumulative HPV infection and future immune protection among HPV-unvaccinated women.

**Methods:**

We tested enrollment sera from participants in the control arm of the Costa Rica Vaccine Trial (n = 488) for HPV16 and HPV18 using GST-L1, VLP-ELISA, and two assays that measure neutralizing antibodies (cLIA and SEAP-NA). With statistical adjustment for sampling, we compared GST-L1 serostatus to established HPV seropositivity correlates and incident cervical HPV infection using odds ratios. We further compared GST-L1 to VLP-ELISA using pair-wise agreement statistics and by defining alternate assay cutoffs.

**Results:**

Odds of HPV16 GST-L1 seropositivity increased with enrollment age (OR = 1.20 per year, 95%CI 1.03-1.40) and lifetime number of sexual partners (OR = 2.06 per partner, 95%CI 1.49-2.83), with similar results for HPV18. GST-L1 seropositivity did not indicate protection from incident infection over 4 years of follow-up (HPV16 adjusted OR = 1.72, 95%CI 0.95-3.13; HPV18 adjusted OR = 0.38, 95%CI 0.12-1.23). Seroprevalence by GST-L1 (HPV16 and HPV18, respectively) was 5.0% and 5.2%, compared to 19.4% and 23.8% by VLP-ELISA, giving positive agreement of 39.2% and 20.8%. Lowering GST-L1 seropositivity cutoffs improved GST-L1/VLP-ELISA positive agreement to 68.6% (HPV16) and 61.5% (HPV18).

**Conclusions:**

Our data support GST-L1 as a marker of cumulative HPV infection, but not immune protection. At lower seropositivity cutoffs, GST-L1 better approximates VLP-ELISA.

## Background

Persistent infection with oncogenic types of human papillomavirus (HPV) is a necessary cause of virtually all cervical cancers [1] and some anogenital and oropharyngeal cancers. Together, HPV types 16 and 18 cause 70% of cervical cancers and 90% of HPV-associated anogenital and oropharyngeal cancers [2].

Measurement of HPV infection is complex. HPV DNA testing using exfoliated cervical cells is the reference standard for identifying current cervical infection, but most infections revert to DNA negativity within 1–2 years [3]. Thus, HPV DNA testing does not reflect past infections that have cleared. Cell mediated, particularly local mucosal, immune responses and generation of serum neutralizing antibodies to the L1 major capsid protein are often detected after infection [4]. These L1 antibodies better reflect both past and present HPV infection (here termed “cumulative infection”), but L1 antibodies are detectable in only about half of women within 18 months of a positive HPV DNA test [5]. Naturally acquired immunity is partially protective against newly detected type-specific HPV infection, though protection by vaccination is much more complete [6-8].

Serological responses to HPV L1 commonly feature as exposures and stratifying variables in epidemiological studies, and are measures of immunogenicity that serve as presumptive correlates of protection in vaccine trials [9]. Several biologically and technically different assays are used to measure type-specific humoral immune responses to HPV L1 capsids. The virus-like particle (VLP)-based enzyme-linked immunosorbent assay (VLP-ELISA) is an established marker of cumulative HPV infection that detects neutralizing and non-neutralizing binding antibodies [10,11]; the competitive Luminex-based immunoassay (cLIA) measures antibodies that compete for binding by pseudovirion-neutralizing monoclonal antibodies (V5 epitope for HPV16-L1; J4 for HPV18-L1) [12]; and the secreted alkaline phosphatase L1/L2 pseudovirion neutralization assay (SEAP-NA) measures overall neutralizing potential against HPV infection [13]. In 2001, a glutathione S-transferase (GST)-L1 fusion protein-based ELISA was developed [14], which was subsequently transferred to a fluorescent bead-based multiplex format [15]. The GST-L1 assay measures both neutralizing and non-neutralizing antibodies to HPV L1 [16], most probably assembled to pentamers [14].

The GST-L1 assay can detect antibodies to up to 100 different antigens simultaneously, has been scaled up for large studies, requires a small specimen volume, and offers a low cost alternative to other assays [15]. Increasingly, the GST-L1 is being used in epidemiology to measure seropositivity to various HPV types and proteins, including L1 of HPV16 and HPV18. These studies largely focus on cancer etiology [17-20] and HPV natural history [21-24]. It is believed that the GST-L1 assay measures cumulative HPV infection and not immune protection, as it does not distinguish between neutralizing and non-neutralizing antibodies. Only an early ELISA-based version of the GST-L1 has been directly compared to the VLP-ELISA [14], while the multiplex GST-L1 has been compared to a VLP multiplex immunoassay [25]. Published data allowing comparison of GST-L1 with neutralization assays and cLIA are few [25-27].

In this study, in the context of naturally acquired HPV infection and immunity, we evaluated whether the GST-L1 assay measures cumulative HPV16/18 infection and/or future immune protection, and directly compared GST-L1 to VLP-ELISA, cLIA, and SEAP-NA.

## Methods

### Study population

Our study population was sampled from the control (HPV-unvaccinated) arm of the Costa Rica Vaccine Trial (CVT), which has been described in detail [28]. The control arm comprised 3,736 women aged 18–25 in Guanacaste, Costa Rica who were followed annually for 4 years, providing a serum sample at each visit. For sexually experienced women, exfoliated cervical cells were also collected during a pelvic exam and used to test for HPV DNA infection at each visit. The CVT protocol was approved by the institutional review boards of the U.S. National Cancer Institute and the Costa Rican INCIENSA, and all participants signed IRB-approved informed consent forms.

**Figure 1:**
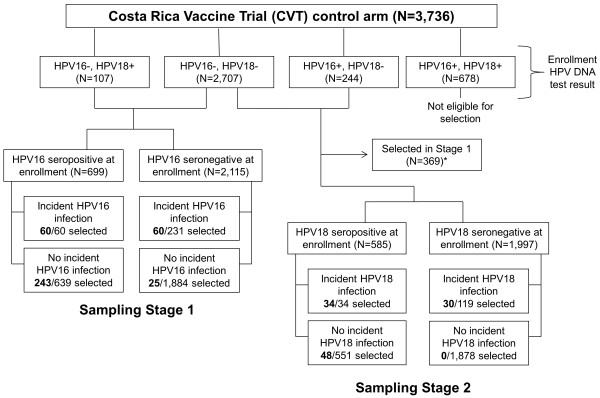
**Two-stage stratified random sampling scheme detailing selection of 500 women from the control (HPV-unvaccinated) arm of the Costa Rica Vaccine Trial (CVT).** Legend: Stage 1 selected 388 women who were HPV16 DNA-negative at the cervix at enrollment, with sampling stratified by HPV16 serostatus at enrollment and HPV16 incident infection status over 4 years of follow-up. Stage 2 augmented the sample using analogous variables for HPV18, selecting 112 women who were HPV18 DNA-negative at enrollment. Twelve women for whom an assay failed were then excluded for analysis. The combined (Stage 1 + Stage 2) sample was weighted to represent all women in the CVT control arm who were HPV16 DNA-negative, HPV18 DNA-negative, or both at enrollment. *For Sampling Stage 2, the women selected in Stage 1 were not eligible to be selected again. Thus, 369 women were removed from the pool of eligible women in Stage 2; this group includes all women selected in Stage 1 who were not HPV18 DNA positive at enrollment (N = 388 selected in Stage 1 – 19 HPV18 DNA-positives = 369).

All women in the CVT control arm were tested at enrollment for HPV16/18 DNA infection at the cervix and for HPV16/18 serum antibodies using VLP-ELISA. Using these results, we sampled 500 women from the CVT control arm using a two-stage stratified random sampling scheme (Figure 1). Stage 1 selected for the HPV16 analysis, which required HPV16 DNA negativity at enrollment, and Stage 2 augmented the sample for the HPV18 analysis, which required HPV18 DNA negativity at enrollment. In Stage 1, we selected 388 of the 2,814 women who were HPV16 DNA negative at enrollment as previously described [29]. Briefly, sampling was stratified by enrollment HPV16 VLP-ELISA serology result (i.e., seropositive or seronegative) and HPV16 incident infection status over 4 years of follow-up, and was designed to ensure sufficient representation from women with positive serology and incident infections. In Stage 2, sampling was stratified by the equivalent variables for HPV18, and with a similar strategy we further sampled 112 women from the 2,582 women who were HPV18 DNA negative at enrollment and not selected in Stage 1.

We performed the GST-L1, VLP-ELISA, and cLIA for HPV16 and HPV18 using enrollment serum samples from the total (N = 500) sample, and the more laborious and costly SEAP-NA for HPV16 on the Stage 1 sample only (N = 388). We assessed assay reproducibility as measured by the intraclass correlation coefficient (ICC) and coefficient of variation among seropositives (CV) for GST-L1, cLIA, and SEAP-NA using 25 blind duplicate specimens included in each batch. We excluded twelve women with failed assays, giving a final sample of 488 women.

We tested cervical samples collected annually from each woman for 4 years for HPV16/18 DNA, and used the results to determine HPV16/18 incident infection status. Detection of HPV16 DNA at any visit for a woman who was HPV16 DNA negative at enrollment constituted an incident infection, and the analogous HPV18 variable was similarly constructed. Though we refer to newly detected infections as incident, we cannot distinguish between a true incident infection, a reactivated latent infection, and a missed prevalent infection.

### GST-L1 multiplex serology

Antibodies to L1 proteins of HPV16 and HPV18 were detected simultaneously by the GST-L1 multiplex assay as previously described [15,30]. Diluted sera were incubated with sets of glutathione casein-coated, fluorescence-labeled, spectrally distinct polystyrene beads, which were loaded with GST-L1-tag fusion proteins. Bound antibodies were detected with anti-human IgG secondary antibody and streptavidin-R-phycoerythrin. Antibody levels for each serum sample are thus expressed as median fluorescence intensity (MFI) of at least 100 beads per color (i.e., antigen) per serum, calculated by subtracting the background fluorescence as determined by a negative control (i.e., no serum) from the raw MFI, then subtracting the MFI of GST-L1-tag (i.e., the fusion protein domains without viral antigen) from the MFI of the specific antigen. Seropositivity cutoffs were determined as previously described [31,32], giving 400 MFI for HPV16 and HPV18.

### VLP-ELISA

The VLP-ELISA was performed at GSK Biologicals as previously described [10]. Briefly, serial dilutions of serum samples and standards were added to ELISA microtiter plates coated with HPV VLPs. A peroxidase-conjugated anti-human polyclonal antibody was added, followed by enzyme substrate and chromogen. Reactions were stopped, and optical density (OD) at 620 nm (background) was subtracted from OD at 450 nm. Antibody levels in ELISA units (EU)/mL were calculated by interpolating OD values from the standard curve, averaging the calculated concentrations from all dilutions that fell within the working range of the reference curve. Seropositivity cutoffs were calculated as 3 standard deviations above the geometric mean titers taken from two groups of known HPV-negative individuals [10,33], giving 8 EU/mL for HPV16 and 7 EU/mL for HPV18.

### cLIA

The multiplex cLIA was performed and cutoffs were determined at PPD Vaccines and Biologicals as previously described [12,33]. Laboratory suggested seropositivity cutoffs were 20 and 24 mMU/mL for HPV16 and HPV18, respectively.

### SEAP-NA

The HPV16 SEAP-NA was performed in duplicate as previously described at the HPV Immunology Laboratory, SAIC-Frederick, Inc. [13,34]. The seropositivity cutoff was set as 3 standard deviations over HPV DNA and seronegatives, giving 25.1 (titer).

### HPV DNA testing

Detection of HPV DNA at the cervix and genotyping was conducted at DDL Diagnostic Laboratory as previously described [35,36]. Briefly, SPF10 primer sets were used to PCR-amplify extracted DNA, then to identify the HPV genotype of SPF10-DEIA-positive samples by reverse hybridization on a line probe assay (LiPA; SPF10-DEIA/HPVLiPA25, version 1; Labo Bio-Medical Products), which detects 25 HPV genotypes. The sensitivity of HPV detection for HPV16 and HPV18 was improved via PCR with type-specific primer sets for specimens testing SPF10-DEIA positive but LiPA25 HPV16 and/or HPV18 negative.

### Statistical methods

We developed a method to calculate sampling weights, which has been described in detail by Li et al. (under revision). Briefly, we first calculated standard inverse probability weights (IPWs) for women selected in both stages by dividing the eligible population in each cell by the number of individuals selected (Figure 1). We then applied two adjustments to make the sample correctly represent the population of women who were both HPV16 and HPV18 DNA negative at enrollment (denoted by P_16/18_). First, we increased the weights for the 369 women selected in Stage 1 who were part of P_16/18_, as they were not eligible for selection in Stage 2. Second, we decreased the weights for women selected in either stage who were part of P_16/18_, as their IPWs were calculated to represent the same population. In other words, without this adjustment, the population P_16/18_ is represented twice. Our final weights allow the sample of 488 women to represent the 3,058 women in the CVT control arm who were HPV16 DNA negative, HPV18 DNA negative, or both at enrollment. We used these weights in all analyses with the exception of HPV16 SEAP-NA analyses, which included only the Stage 1 sample and therefore required standard IPWs.

We performed each analysis separately for HPV16 and HPV18. Type-specific DNA negativity at enrollment was required for inclusion in the analysis for each HPV type, resulting in inclusion of 467 women in the HPV16 analysis (weighted N = 2,786) and 477 women in the HPV18 analysis (weighted N = 2,979). We first examined distributions of GST-L1 antibody reactivity (minimum, maximum, geometric mean, and quartiles) among all subjects, among GST-L1 seropositives, and among women with and without an incident type-specific infection over follow-up.

To assess the utility of GST-L1 as a marker of cumulative HPV infection, we calculated odds ratios for GST-L1 seropositivity based on two established correlates of HPV seropositivity, age at enrollment (an HPV seropositivity correlate among younger women) and lifetime number of sexual partners at enrollment (hereafter referred to as “number of partners”) [37,38]. To depict these relationships, we plotted seroprevalence by age and number of partners for a) GST-L1 at the laboratory cutoff, b) VLP-ELISA at the laboratory cutoff, c) GST-L1 at alternate cutoffs that maximized agreement with VLP-ELISA as described below, d) cLIA at the laboratory cutoff, and e) SEAP-NA at the laboratory cutoff.

To address whether GST-L1 indicates immune protection from future HPV infection, we calculated odds ratios for incident cervical HPV infection based on GST-L1 seropositivity at the laboratory cutoff and quintiles of GST-L1 antibody reactivity, with adjustment for number of partners.

We examined agreement between GST-L1 and VLP-ELISA, cLIA, and SEAP-NA using percent agreement, positive agreement [39], and kappa statistics. For pair-wise agreement statistics, we used both laboratory suggested and “alternate” seropositivity cutoffs, which were calculated to assess the degree to which pair-wise discordance between GST-L1 and VLP-ELISA is due to non-calibration of seropositivity cutoffs. Using VLP-ELISA as the alloy standard, we defined alternate GST-L1 and VLP-ELISA seropositivity cutoffs to maximize positive agreement and separately Youden index (sensitivity + specificity-1). Specifically, we calculated the statistic of interest (e.g. positive agreement) based on each possible cutoff for the assay in question, and identified the cutoff that gave the highest value of the statistic. We present data using alternate GST-L1 cutoffs only, as these gave better results.

We conducted analyses in R version 2.15.1 (generation of weights and calculation of odds ratios) and Stata 11 (College Station, TX, all others). Statistical significance was assessed at α = 0.05.

## Results

### Assay reproducibility

For GST-L1, ICCs based on our data were 99.3% for HPV16 and 96.9% for HPV18, and internal laboratory data gave CVs of 13.4% for HPV16 and 13.7% for HPV18. For VLP-ELISA, extensive testing was performed for the CVT and overall reproducibility data were calculated; the VLP-ELISA has a mean CV of 12.3% in the CVT [10]. For cLIA, ICCs based on our data were 93.1% and 92.2%, and CVs were 10.3% and 10.0%. For HPV16 SEAP-NA, the ICC and CV based on our data were 95.4% and 12.9%, respectively.

### HPV16

**Figure 2:**
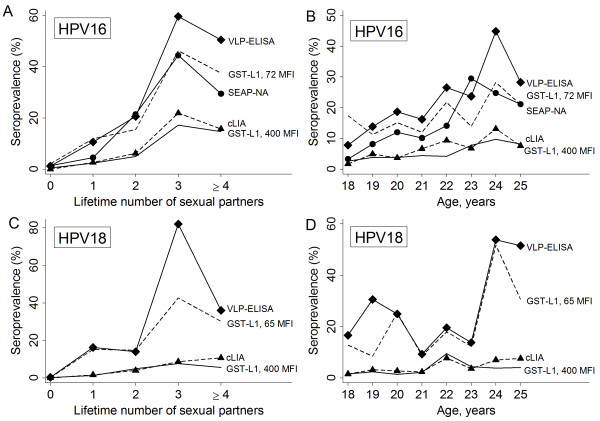
**Sampling-adjusted seroprevalence measured by different assays, stratified by lifetime number of sexual partners and age at enrollment.** Legend: Seroprevalence vs. number of partners (Panels **A** and **C**) and age (Panels **B** and **D**) is shown for HPV16 (Panels **A-B**) and HPV18 (Panels **C-D**). Lines represent seroprevalence by GST-L1 at the laboratory suggested seropositivity cutoff (solid line), GST-L1 at alternate cutoffs (dashed line), VLP-ELISA (solid line with diamond markers), cLIA (dashed line with triangle markers), and SEAP-NA (solid line with circle markers, HPV16 only). Alternate GST-L1 cutoffs were calculated to maximize agreement with VLP-ELISA (see Statistical Methods). Odds of GST-L1 seropositivity at the laboratory cutoff increased with each year of age for HPV16 (OR 1.20, 95% CI 1.03-1.40) and HPV18 (OR 1.20, 95% CI 1.04-1.38). Similarly, odds increased with each sexual partner for HPV16 (OR 2.06, 95% CI 1.49-2.83) and HPV18 (OR 1.59, 95% CI 1.18-2.15). For HPV18, one observation with a large weight was excluded for figure only.

For HPV16, GST-L1 antibody reactivity ranged from 0 to 5979 MFI; ranges among subgroups defined by seropositivity and incident infection status are shown in Table 1. Overall seroprevalence was 5.0% by GST-L1 at the laboratory cutoff, 19.4% by VLP-ELISA, 5.8% by cLIA, and 13.8% by SEAP-NA. Stratified by lifetime number of sexual partners at enrollment, seroprevalence by GST-L1 ranged from 0.5% among self-reported virgins to 17.2% among women with 3 partners (Figure 2A), and odds of seropositivity increased significantly with number of partners (OR 2.06 per partner, 95% CI 1.49-2.83). Similarly, GST-L1 seroprevalence ranged from 2.6% among 18-year-olds to 9.8% among 24-year-olds (Figure 2B), and odds of seropositivity increased significantly with age (OR 1.20 per year, 95% CI 1.03-1.40). Within categories of number of partners and age, GST-L1 seroprevalence at the 400 MFI (laboratory suggested) cutoff was very similar to cLIA but lower than VLP-ELISA and SEAP-NA (Figure 2A-B).

**Table 1:** Sampling-adjusted data describing antibody levels measured by the GST-L1 assay among HPV-unvaccinated women in the Costa Rica Vaccine Trial

**HPV type**		**Min (MFI)**	**Max (MFI)**	**Geometric mean (MFI)**	Q1* (MFI)	Q2*, median (MFI)	Q3* (MFI)
HPV 16	All women (N = 2,786)	0	5979	19	5	30	67
	Seropositive women (N = 140)	415	5979	902	570	777	1329
	HPV16 infection (N = 211)	0	1709	28	8	40	104
	No HPV16 infection (N = 2575)	0	5979	18	5	30	66
HPV18	All women (N = 2,979)	0	2849	17	3	26	57
	Seropositive women (N = 155)	416	2849	677	528	528	930
	HPV18 infection (N = 196)	0	2696	17	9	24	33
	No HPV18 infection (N = 2783)	0	2849	17	3	26	57

*Q1, Q2, and Q3 refer to the first, second, and third quartiles, respectively, of the sampling-adjusted distribution of antibody levels.

“Infection” refers to incident infection over 4 years of follow-up. Analyses are based on 467 women for HPV16 and 477 women for HPV18; displayed sample sizes are sampling-adjusted.

Seropositivity by GST-L1 at enrollment did not indicate lower risk of incident HPV16 infection over follow-up with adjustment for number of partners (adjusted OR 1.72, 95% CI 0.95-3.13, Table 2). Similarly, higher quintiles of GST-L1 antibody reactivity did not indicate immune protection (data not shown).

**Table 2:** Sampling-adjusted odds of incident infection based on GST-L1 seropositivity

**HPV type**		**Incident type-specific HPV infection**		aOR* (95% CI)
		**No, N (%)**	**Yes, N (%)**	
HPV16	GST-L1 –	2,455 (92.8)	191 (7.2)	Reference
	GST-L1 +	120 (85.7)	20 (14.3)	1.72 (0.95-3.13)
HPV18	GST-L1 –	2,633 (93.2)	191 (6.8)	Reference
	GST-L1 +	151 (97.1)	5 (2.9)	0.38 (0.12-1.23)

*Odds ratio adjusted for lifetime number of sexual partners at enrollment, in categories of 0, 1, 2, 3, and ≥4.

Analyses are based on 467 women for HPV16 and 477 women for HPV18; displayed sample sizes are sampling-adjusted.

**Figure 3:**
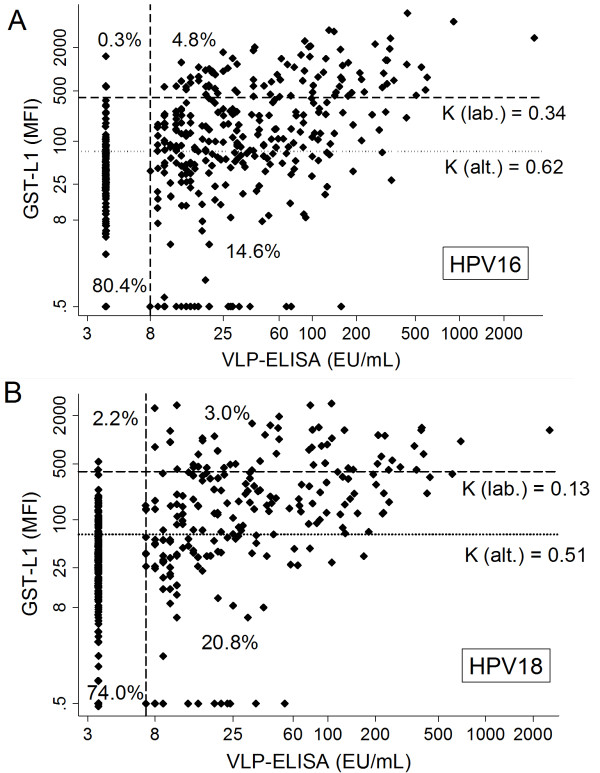
**Antibody levels as measured by GST-L1 vs. VLP-ELISA among HPV-unvaccinated women.** Legend: Antibody levels are displayed on a logarithmic scale for HPV16 (Panel **A**) and HPV18 (Panel **B**). Dashed lines represent laboratory suggested seropositivity cutoffs, and dotted lines represent alternate GST-L1 cutoffs calculated to maximize agreement with VLP-ELISA (see Statistical Methods). K refers to the kappa statistic, calculated separately at the laboratory (lab.) and alternate (alt.) cutoffs. Points that appear to form a line reflect assay limits of detection. Percentages displayed are sampling-adjusted and are based on laboratory cutoffs.

At the laboratory suggested cutoffs, overall agreement between GST-L1 and VLP-ELISA was 85.1%, and positive agreement was 39.2% (Table 3). Discordance was largely due to a substantial proportion (14.6%) of women who were seropositive by VLP-ELISA but seronegative by GST-L1 (Table 3 and Figure 3A). To assess how much GST-L1/VLP-ELISA pair-wise discordance was related to differences in seropositivity cutoffs, we examined alternate cutoffs to maximize positive agreement and, separately, Youden index. Lowering the GST-L1 cutoff from 400 to 72 MFI maximized both of these and reduced the overall percentage of discordant samples, increasing overall agreement to 88.7% and positive agreement to 68.6% (Table 3 and Figure 3A). At this cutoff, overall seroprevalence by GST-L1 increased to 16.6%, and estimates of seroprevalence stratified by age and number of partners were more similar to those by VLP-ELISA and SEAP-NA (Figure 2A-B). The proportion of samples negative by GST-L1 but positive by VLP-ELISA decreased, while inevitably the proportion of samples positive by GST-L1 but negative by VLP-ELISA increased (Table 3 and Figure 3A).

**Table 3:** Sampling-adjusted agreement between GST-L1 and VLP-ELISA at GST-L1 laboratory suggested (400 MFI) and alternate cutoffs

**HPV type**	**GST-L1 cutoff (MFI)**		**GST-L1 + (N)**	**GST-L1 – (N)**	**Percent agreement**	**Positive agreement**	**Kappa**
HPV 16	400	VLP-ELISA +	133	407	85.1%	39.2%	0.34
		VLP-ELISA –	7	2,239			
	72	VLP-ELISA +	344	197	88.7%	68.6%	0.62
		VLP-ELISA –	118	2,128			
	400	cLIA +	64	98	93.7%	42.1%	0.39
		cLIA –	76	2,547			
	72	cLIA +	122	40	86.4%	39.1%	0.33
		cLIA –	339	2,284			
	400	SEAP-NA +	92	296	86.8%	33.1%	0.27
		SEAP-NA –	77	2,349			
	72	SEAP-NA +	199	189	79.9%	41.3%	0.30
		SEAP-NA –	377	2,049			
HPV 18	400	VLP-ELISA +	90	618	77.1%	20.8%	0.13
		VLP-ELISA –	65	2,206			
	65	VLP-ELISA +	403	305	83.1%	61.5%	0.51
		VLP-ELISA –	199	2,072			
	400	cLIA +	43	67	94.0%	32.1%	0.29
		cLIA –	113	2,757			
	65	cLIA +	100	9	82.8%	28.2%	0.23
		cLIA –	502	2,367			

Analyses are based on 467 women for HPV16 and 477 women for HPV18; displayed sample sizes are sampling-adjusted. Alternate GST-L1 cutoffs were calculated to maximize agreement with VLP-ELISA (see Statistical Methods).

Positive agreement was relatively low between GST-L1 and cLIA (42.1%, Table 3) and between GST-L1 and SEAP-NA (33.1%). At the 72 MFI cutoff for GST-L1, which maximized agreement with ELISA, positive agreement with cLIA was slightly lower (39.1%) and with SEAP-NA was marginally higher (41.3%).

### HPV18

For HPV18, GST-L1 antibody reactivity ranged from 0 to 2849 MFI; ranges among subgroups are shown in Table 1. Overall seroprevalence was 5.2% by GST-L1, 23.8% by VLP-ELISA, and 3.7% by cLIA. Stratified GST-L1 seroprevalence estimates ranged from 0.2% among self-reported virgins to 7.2% among women with 3 partners (Figure 2C), and odds of seropositivity increased with number of partners (OR 1.59 per partner, 95% CI 1.18-2.15). By age, seroprevalence was lowest among 20-year-olds (1.4%, Figure 2D) and highest among 22-year-olds (9.5%), and odds of seropositivity increased with age (OR 1.20 per year, 95% CI 1.04-1.38). Within categories of number of partners and age, GST-L1 seroprevalence at the 400 MFI cutoff was similar to cLIA but lower than VLP-ELISA (Figure 2C-D).

Enrollment seropositivity by GST-L1 was not significantly associated with lower risk of incident infection (adjusted OR 0.38, 95% CI 0.12-1.23). Examining quintiles of GST-L1 antibody reactivity did not alter (data not shown).

At the laboratory suggested cutoffs, overall agreement between GST-L1 and VLP-ELISA was 77.1%, and positive agreement was 20.8% (Table 3). About one-fifth of women were seropositive by VLP-ELISA but seronegative by GST-L1 (Table 3 and Figure 3B). Lowering the GST-L1 cutoff from 400 to 65 MFI maximized both positive agreement and Youden index; positive agreement nearly tripled to reach 61.5% and overall agreement increased marginally to 83.1% (Table 3 and Figure 3B). At this cutoff, overall seroprevalence by GST-L1 was 20.2%, and stratified estimates of seroprevalence were similar to those by VLP-ELISA (Figure 2C-D). As expected, the proportion of samples positive by GST-L1 but negative by VLP-ELISA increased at the lower cutoff (Table 3 and Figure 3B).

Positive agreement between GST-L1 and cLIA was low (32.1%, Table 3) and slightly lower at the 65 MFI cutoff that maximized agreement with ELISA (28.2%).

## Discussion

GST-L1 multiplex serology measures antibodies to many HPV types at high-throughput and low cost, and is increasingly being used in HPV seroepidemiology. Our results support the GST-L1 assay as a marker of cumulative HPV16/18 infection, but not of future immune protection. Direct comparison between the bead-based GST-L1 and VLP-ELISA suggested only modest correlation between these two tests designed to measure cumulative HPV infection, but much of the observed lack of agreement was explained by differences between the seropositivity cutoffs for each assay.

Seroprevalence of HPV is known to increase with lifetime number of sexual partners and with age among young women [37,38]. We noted increasing odds of GST-L1 seropositivity with each, supporting GST-L1 as a marker of cumulative HPV infection. This is consistent with documented associations between GST-L1 seropositivity and ever/never sexual activity, age at sexual debut, and lifetime number of sexual partners [22,31,40]. VLP-ELISA is an established HPV exposure marker [11], and we observed moderate concordance between GST-L1 and VLP-ELISA. A previous study compared GST-L1 to a VLP multiplex immunoassay, which is biologically similar but technically different from our VLP-ELISA. The authors found percent agreement of 66% and 62% between the assays for HPV16 and HPV18, respectively [25], lower than our figures of 85% and 77%.

In contrast, we found that GST-L1 serology is not a good measure of immune protection from future HPV infection; this result differs from findings for other assays. Our group previously found that HPV16 cLIA seropositivity is associated with a lower risk of incident infection, while for VLP-ELISA and SEAP-NA, the highest seropositive tertile of antibody levels indicates protection [7,41]. Further, positive agreement between GST-L1 and both cLIA and SEAP-NA, which measure neutralizing antibodies that are believed to confer immune protection, was poor. This occurred despite similar seroprevalence estimates by GST-L1 and cLIA. Though one previous study found no difference in type-specific GST-L1 seropositivity between women with and without an incident HPV DNA infection [23], these authors and another group found associations suggesting immune protection measured by GST-L1 when using extreme or stringently defined viral outcomes [23,42].

We do not always expect different HPV serological assays to have high pair-wise concordance, because they do not measure equivalent aspects of the immune response and their seropositivity cutoffs are not calibrated to one another. Both GST-L1 and VLP-ELISA are capable of measuring polyclonal responses, and neither is restricted to measuring neutralizing antibodies. Though the assays are similar biologically, our results demonstrate that differences in seropositivity cutoffs between GST-L1 and ELISA produce substantial pair-wise discordance. Our group previously found positive agreement for HPV16 among unvaccinated women to be 45% for VLP-ELISA/cLIA, 55% for VLP-ELISA/SEAP-NA, and 68% for cLIA/SEAP-NA [29]. In the present study, positive agreement between GST-L1 and other assays at laboratory cutoffs did not exceed 42%. One study of the early GST-based ELISA found substantial (kappa = 0.62) agreement with the VLP-based ELISA [14]. This is higher than our result (kappa = 0.34), likely because the study included a high proportion of VLP-ELISA seropositives and used a GST-L1 cutoff that gave equal numbers of seropositive samples by the two assays.

The laboratory suggested seropositivity cutoff is lower for VLP-ELISA than GST-L1 compared to their respective overall distributions over the entire range of antibody levels. The higher GST-L1 cutoff gives high specificity and seroprevalence estimates that are robust to small cutoff adjustments [43], but it produces pair-wise discordance with VLP-ELISA. In our study, lack of correspondence between cutoffs explained a large portion of pair-wise discordance between GST-L1 and VLP-ELISA, as positive agreement improved from 20-40% to 60-70% with adjustment of GST-L1 cutoffs. However, use of a lower GST-L1 cutoff increases the proportion of samples positive by GST-L1 but negative by VLP-ELISA; the samples that enter this category are visible between the reference lines for the laboratory and alternate cutoffs in the upper-left quadrants of Figure 3A-B. These discordant samples at a lower cutoff may result from nonspecific background in the GST-L1 assay [25], which complicates distinction between seronegative and low positive results. For most serologic assays, including GST-L1, seroprevalence estimates and assay sensitivity and specificity depend strongly on the seropositivity cutoff. Therefore, population studies of naturally acquired cumulative HPV infection that use GST-L1 should be interpreted in light of the cutoff employed.

Our results aid interpretation and design of research involving HPV seroepidemiology. For example, HPV16 seroprevalence has been reported as 13.0% and 7.1% in the United States and German general populations, respectively [21,44]. However, as VLP-ELISA was used in the U.S. study and GST-L1 in the German study, the discrepancy could reflect assay differences in addition to differences in study design or true population level differences in HPV16 exposure. When choosing an assay, investigators should consider implications for comparability to other studies, specify whether they aim to measure HPV cumulative infection or immune protection, and address discrepancies that may arise due to lack of calibration between assay cutoffs. While using laboratory suggested cutoffs is important for consistency across studies using the same assay, additional analyses using alternate cutoffs may enable approximate comparison across studies using different assays. Importantly, however, comparisons are hampered by issues beyond non-calibration of seropositivity cutoffs, and would be aided by use of quality control panels and development of a universal reference standard with a known antibody concentration for many HPV types.

Strengths of our study include a well-defined source population, complete follow-up data, validated laboratory techniques, and assay reproducibility data. One limitation is that we were unable to assess the association of GST-L1 with current cervical infection, because type-specific DNA negativity was required for inclusion in each analysis. The utility of GST-L1 as a cumulative infection marker would be supported by a finding of higher GST-L1 seroprevalence among women with a current cervical infection. This has been reported by some studies [24,31,40] though results have not been entirely consistent [22,23]. Finally, it is unknown whether the same alternate cutoffs calculated in our study would be obtained using other ELISA reagents, such as VLPs, or in other laboratories.

## Conclusions

Our data suggest that the GST-L1 assay is a measure of cumulative HPV infection, but not future immune protection, and that a portion of pair-wise discordance between GST-L1 and VLP-ELISA is explained by lack of calibration between assay seropositivity cutoffs.

Each of the assays studied here has important advantages and disadvantages for use in seroepidemiologic research. When neutralizing responses and immune protection are of interest, SEAP-NA is the most comprehensive measure of neutralization potential but is laborious and costly; cLIA is more efficient but measures only a subset of neutralizing antibodies (i.e., antibodies that bind to one neutralizing epitope).

As a marker of cumulative HPV infection, the GST-L1 assay measures many different HPV types in a high-throughput manner at relatively low cost. However, our data suggest GST-L1 is not appropriate for use as a marker of immune protection from future cervical HPV infection, and that its comparability to cLIA and SEAP-NA is limited. The GST-L1 more closely approximates the commonly used VLP-ELISA at a lower cutoff, and may be an appropriate choice for studies aiming to assess population-level patterns in the epidemiology of cumulative infection with many HPV types.

## Abbreviations

cLIA: Competitive Luminex immunoassay; CV: Coefficient of variation; CVT: Costa Rica Vaccine Trial; ELISA: Enzyme-linked immunosorbent assay; GST: Glutathione S-transferase; HPV: Human papillomavirus; ICC: Intraclass correlation coefficient; MFI: Median fluorescence intensity; OD: Optical density; SEAP-NA: Secreted alkaline phosphatase neutralization assay; VLP: Virus-like particle.

## Competing interests

One or more of the authors is employed by a commercial company. These include: Mark Esser, MedImmune; Katie Matys, PPD Vaccines and Biologics; Wim Quint and Leen-Jan van Doorn, DDL Diagnostic Laboratory. John Schiller and Douglas Lowy report that they are named inventors on U.S. Government-owned HPV vaccine patents that are licensed to GlaxoSmithKline and Merck and for which the National Cancer Institute receives licensing fees. They are entitled to limited royalties as specified by federal law. The other authors declare that they have no conflicts of interest.

## Authors’ contributions

Conception and design: HAR, YL, MP, AG, M. Schiffman, SW, RH, AH, TW, M. Safaeian. Acquisition of data: CP, AG, ACR, M. Schiffman, SW, PG, JS, DL, RH, LAP, AH, M. Safaeian. Serological assays: MP, TJK, ME, KM, WQ, LJvD, LAP, TW. Statistical analysis: HAR, YL, AG, M. Safaeian. Interpretation of data: HAR, YL, MP, M. Schiffman, SW, TJK, DL, LAP, AH, TW, M. Safaeian. Drafting and revision of manuscript: HAR, YL, CP, MP, AG, ACR, M. Schiffman, SW, TJK, PG, JS, DL, ME, KM, WQ, LJvD, RH, LAP, AH, TW, M. Safaeian. All authors read and approved the final manuscript.

## Pre-publication history

The pre-publication history for this paper can be accessed here:

http://www.biomedcentral.com/1471-2334/14/120/prepub
